# Supplementary material for: Predicting Functional Consequences of Recent Natural Selection in Britain
Source: Mol Biol Evol. 2024 Mar 11;41(3):msae053. doi: 10.1093/molbev/msae053 (PMC10962637; doi:10.1093/molbev/msae053)
Supplement: msae053_Supplementary_Data [file msae053_supplementary_data.zip › Supplementary Figures and Table 1.pdf]

Supplementary Materials

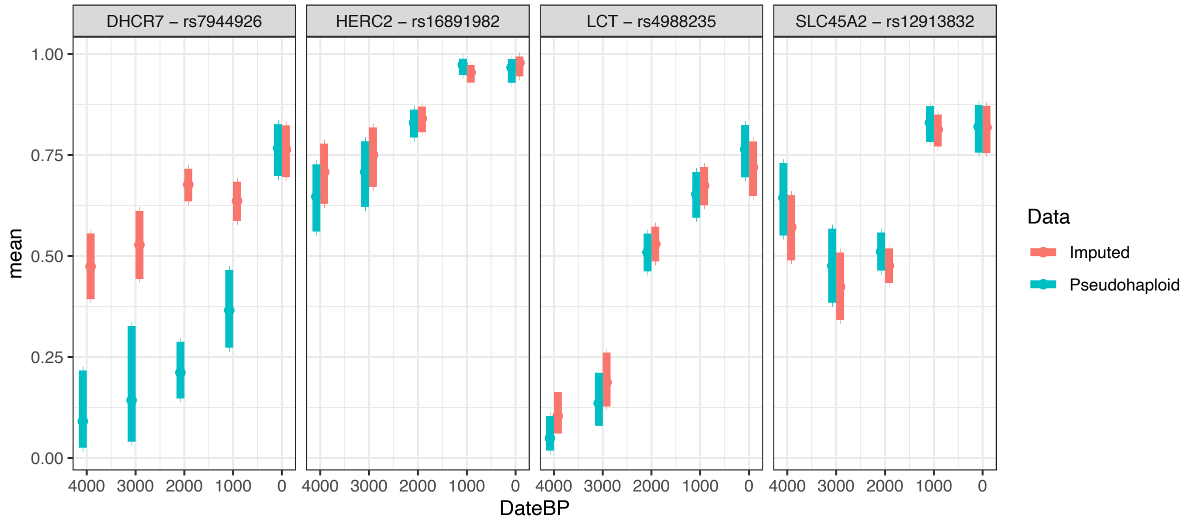

Supplementary Figure 1: Estimated derived allele frequencies and 95% confidence intervals for samples in 1000-year bins for four different strongly selected variants. Pseudohaploid data in blue and imputed diplod data in red. In the first three cases, the imputed allele frequencies are biased towards the present-day frequencies, suggesting that selection signals at these loci would be attenuated.

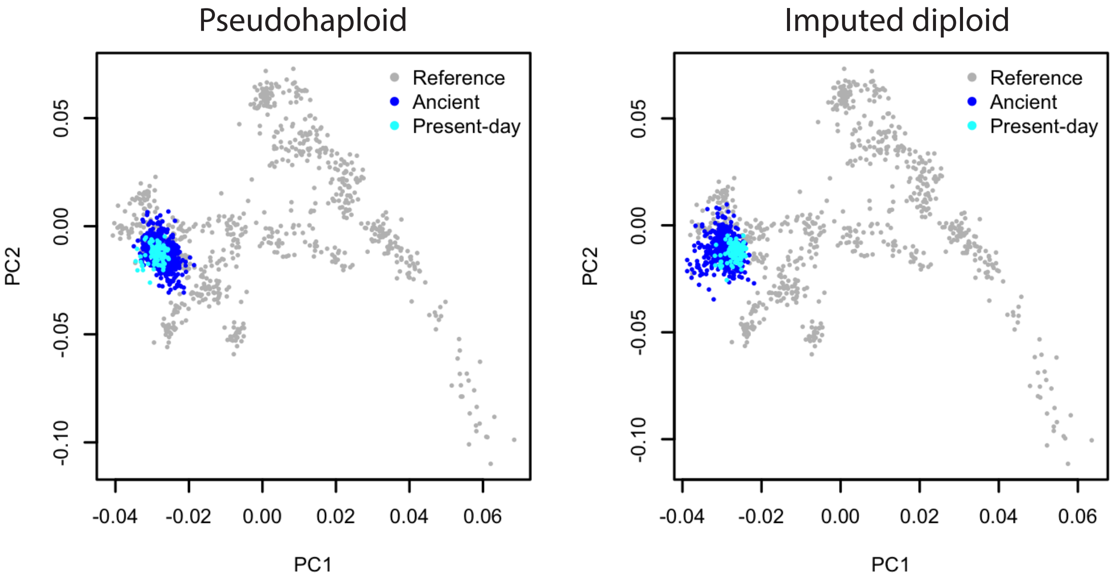

Supplementary Figure 2: PCA plots of ancient (dark blue) and present-day (light blue) samples used in the analysis projected onto principal components defined by 777 present-day West Eurasian samples genotyped on the Human Origins array (see Lazaridis et al. (2014) for details and population labels). In the left panel the ancient samples are pseudohaploid, while in the right panel they are imputed to diploid coverage. Only sites included on the Human Origins array are included.

Chr	Gene	Tissue	R^2	P-value	Beta	GWSS Peak
1	*SLC44A5*	Skin Sun Exposed Lower leg	0.556	4.331e-06	-8.694e-05	Novel
2	*MGAT5*	Lung	0.026	1.805e-05	4.752e-07	*LCT*
2	*TMEM163*	Kidney Cortex	0.457	6.738e-15	1.571e-04	*LCT*
2	*ZRANB3*	Cells Cultured fibroblasts	0.086	6.733e-09	9.010e-06	*LCT*
2	*UBXN4*	Brain Cerebellar Hemisphere	0.091	5.114e-13	1.651e-05	*LCT*
2	*LCT*	Cells EBV-transformed lymphocytes	0.039	3.153e-16	2.497e-05	LCT
2	*MCM6*	Esophagus Muscularis	0.176	6.099e-22	-1.305e-04	*LCT*
2	*DARS*	Skin Not Sun Exposed Suprapubic	0.068	1.170e-06	-2.081e-05	*LCT*
4	*TLR10*	Cells EBV-transformed lymphocytes	0.060	8.518e-05	-1.977e-06	Novel
5	*P4HA2*	Thyroid	0.379	8.541e-05	7.661e-05	*PDLIM4*
5	*PDLIM4*	Brain Cortex	0.235	4.076e-07	-9.183e-05	*PDLIM4*
5	*SLC22A4*	Artery Aorta	0.079	1.327e-05	5.114e-06	*PDLIM4*
5	*SLC22A5*	Cells Cultured fibroblasts	0.446	1.538e-05	-1.003e-04	*PDLIM4*
6	*PRSS16*	Ovary	0.322	4.302e-05	4.963e-05	Novel
6	*CCHCR1*	Spleen	0.564	2.140e-06	8.265e-05	HLA
6	*TCF19*	Brain Cerebellar Hemisphere	0.473	9.591e-05	-5.660e-05	HLA
6	*APOM*	Pituitary	0.072	1.434e-05	-7.049e-06	HLA
6	*C4A*	Brain Cerebellum	0.423	2.226e-05	-5.035e-05	HLA
6	*RNF5*	Colon Transverse	0.274	4.229e-09	-7.185e-05	HLA
6	*AGER*	Nerve Tibial	0.328	7.746e-11	7.944e-05	HLA
11	*FADS1*	Brain Cerebellum	0.460	3.520e-05	8.132e-05	*FADS1*
11	*FADS3*	Brain Cerebellum	0.158	6.401e-05	1.041e-05	*FADS1*
11	*DHCR7*	Small Intestine Terminal Ileum	0.071	1.024e-05	1.358e-05	*DHCR7*
12	*OAS1*	Spleen	0.114	8.517e-05	-8.373e-06	OAS
12	*OAS3*	Cells Cultured fibroblasts	0.391	1.115e-06	-5.666e-05	OAS
17	*NUP85*	Brain Cerebellum	0.638	4.198e-05	9.050e-05	Novel
17	*MRPS7*	Brain Cerebellar Hemisphere	0.413	2.854e-05	-5.346e-05	Novel

Supplementary Table 1: Genes with significant shifts in predicted expression (FDR < 0.05) characterized by the transcriptome-wide selection scan based on UTMOST models. *Tissue* indicates which tissue model was used. Note that this does not mean that the gene did not have significant shifts in other tissues, just that this tissue had the highest UTMOST training R^2. *R2* indicates the UTMOST training R^2 for these tissues. *Beta* indicates the effect size of time on expression levels in the ordinary regression models. *GWSS Peak* indicates the significant (FDR < 0.05) genome-wide selection scan peak indicated in Figure 1 to which each gene corresponds. *MGAT5* did not fit the criteria to be included in the *LCT* peak (its midpoint was not within the bounds of the LCT peak) but overlapped with it, and was thus included under *LCT*.

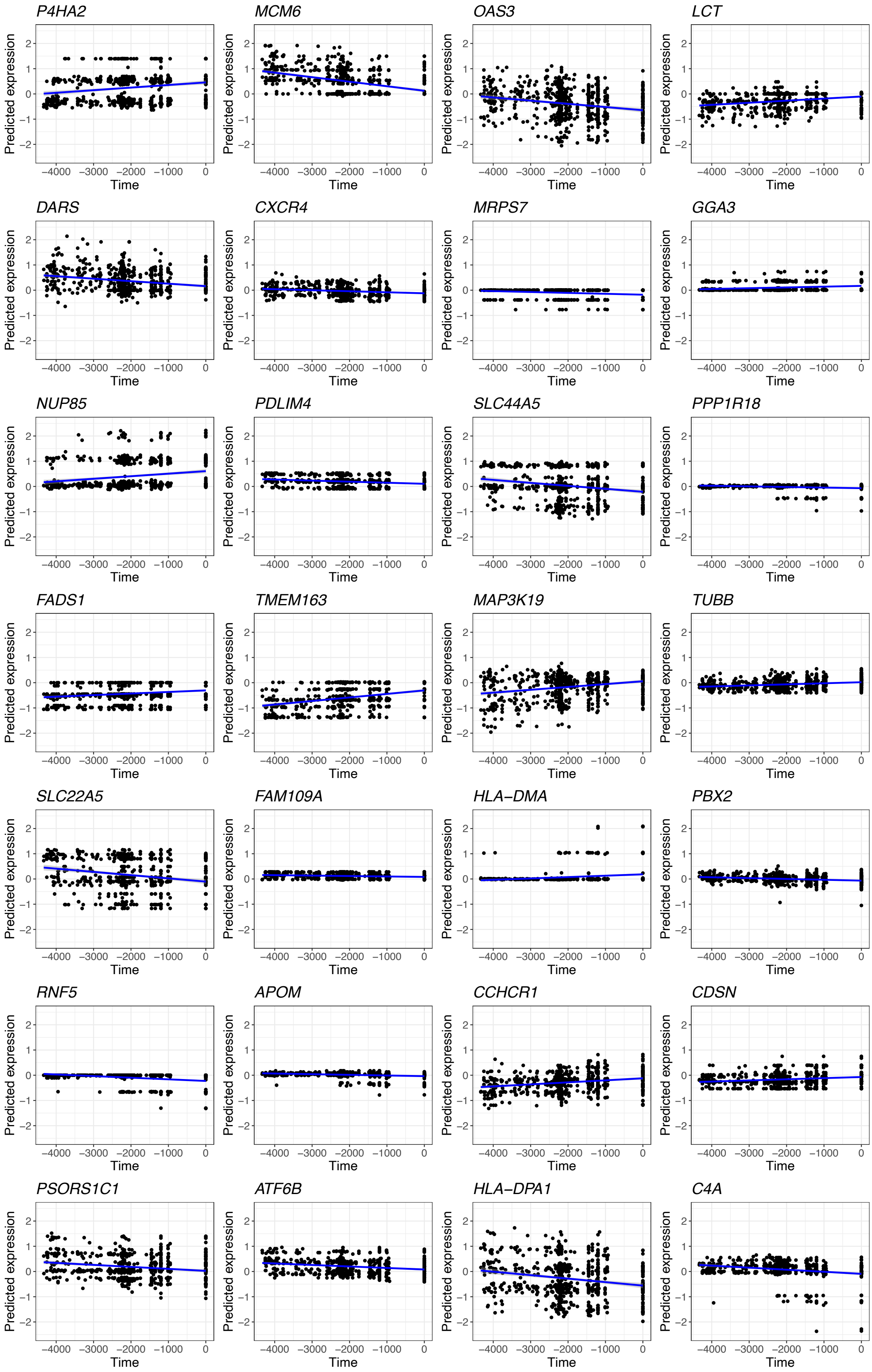

Supplementary Figure 3: Changes in gene expression across time for significant genes. The x-axis indicates time, where 0 represents modern samples. The y-axis indicates predicted normalized expression level. Each point represents a sample.

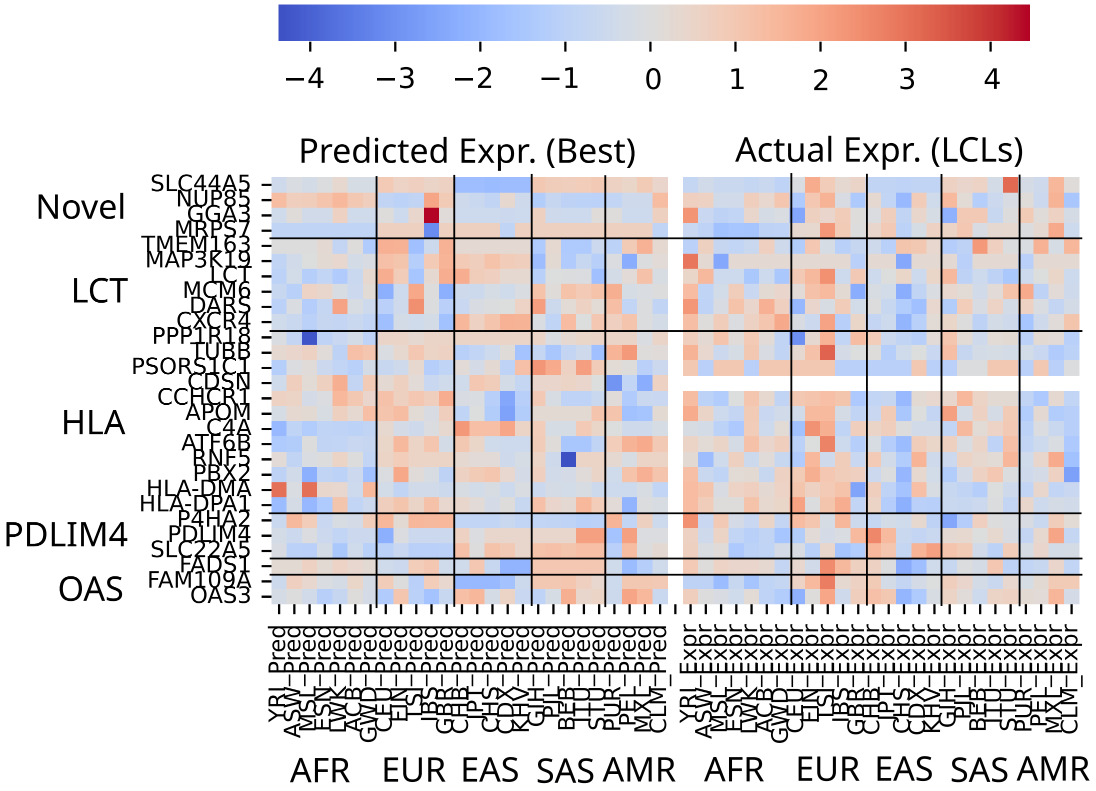

Supplementary Figure 4: Predicted and actual expression of significant genes. Heatmap shows median predicted expression based on JTI models (Zhou et al., 2020) and median actual expression in LCLs (Taylor et al., 2023) for all 1000 Genomes Populations (1000 Genomes Consortium, 2015). Values are normalized so that the standard deviation over individuals equals 1. Genes are organized by locus. Observed and Predicted expression agree more often than expected by chance (p = 0.0053).

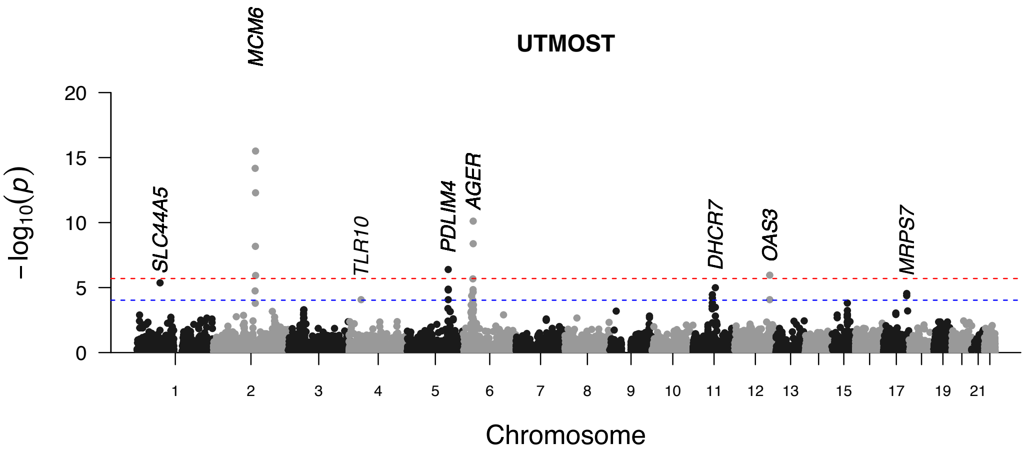

Supplementary Figure 5: Transcriptome-wide scan for selection based on UTMOST models. Each point represents a gene. Blue lines indicate FDR significance ($P < 10^{-4}$) and red lines indicate Bonferroni significance ($P < 4 * 10^{-6}$). The most significant gene at each locus is labeled. All peaks in the JTI scan are replicated in the UTMOST scan.

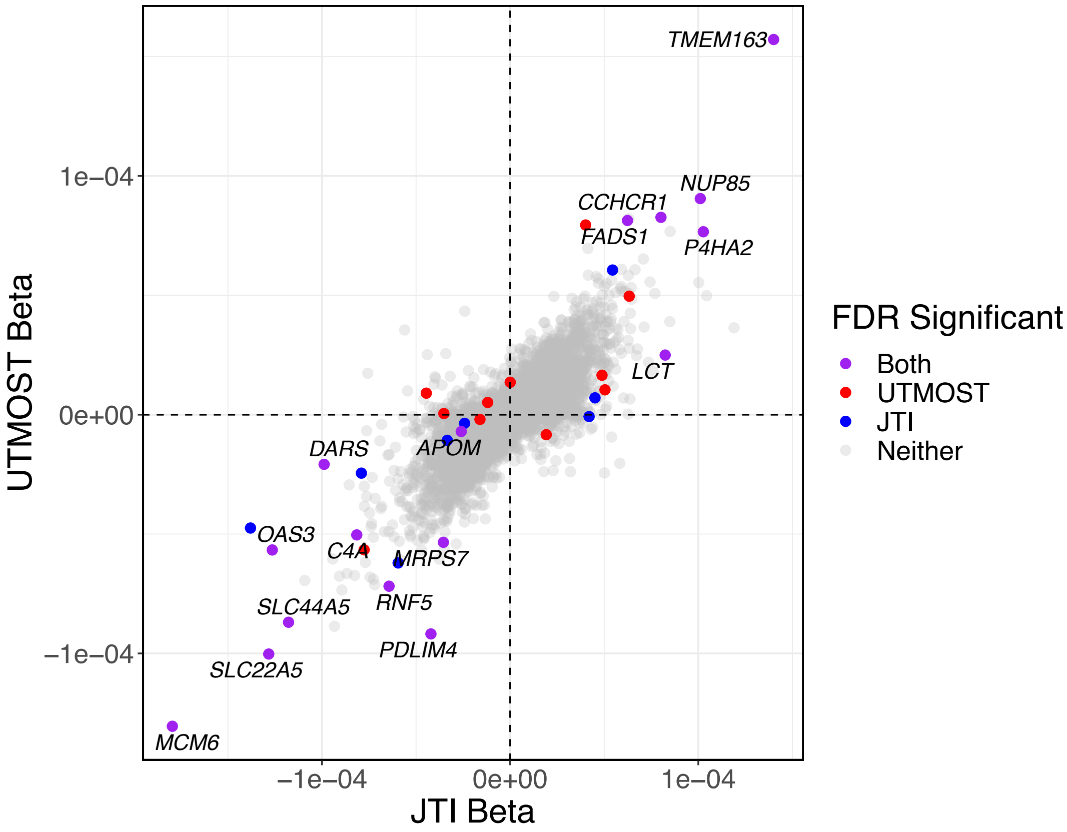

Supplementary Figure 6: Direction of effect predicted by JTI and UTMOST are largely concordant. The x-axis indicates average change in predicted gene expression per year as measured by the beta of time in the transcriptome-wide scan based on JTI models. The y-axis indicates betas of time in the scan based on UTMOST models. The Pearson R^2 is 0.797. Each point represents a gene, with purple points indicating genes that are significant in both analysis, red points indicating those that are only significant in the UTMOST scan, blue the JTI scan, and gray in neither.
